# Health-Related Quality of Life and Functional Status of Post-COVID-19 Patients

**DOI:** 10.3390/ijerph22030338

**Published:** 2025-02-25

**Authors:** Miriã C. Oliveira, Larissa R. Alves, Juliana M. P. Soares, Shayra K. A. Souza, Bruna M. R. Silva, Adriano L. Fonseca, Carlos H. M. Silva, Claudia S. Oliveira, Rodolfo P. Vieira, Deise A. A. P. Oliveira, Iransé Oliveira-Silva, Rodrigo F. Oliveira, Luciana M. M. Sampaio, Vinicius Maldaner, Dante B. Santos, Renata K. Palma, Sergio R. Nacif, Giuseppe Insalaco, Luís V. F. Oliveira

**Affiliations:** 1 Human Movement and Rehabilitation Graduate Program, Evangelical University of Goiás (UniEVANGELICA), Anápolis 75083-515, GO, Brazil; miriacandidaoliveira@gmail.com (M.C.O.); juliana.soares@unievangelica.edu.br (J.M.P.S.); fonseca.luis.adriano@gmail.com (A.L.F.); csantos.neuro@gmail.com (C.S.O.); rodrelena@yahoo.com.br (R.P.V.); deisepyres@gmail.com (D.A.A.P.O.); iranse.silva@unievangelica.edu.br (I.O.-S.); rodrigofranco65@gmail.com (R.F.O.); viniciusmaldaner@gmail.com (V.M.); dantebsantos@gmail.com (D.B.S.); rekellyp@hotmail.com (R.K.P.); 2 Faculty of Medicine, Evangelical University of Goiás (UniEVANGELICA), Anápolis 75083-515, GO, Brazil; lari.ralves@gmail.com (L.R.A.); carloshmendes@unievangelica.edu.br (C.H.M.S.); 3 Health Sciences Graduate Program, Faculty of Medical Sciences of Santa Casa de São Paulo, São Paulo 01224-001, SP, Brazil; 4 Scientific Initiation Program, Evangelical University of Goiás (UniEVANGELICA), Anápolis 75083-515, GO, Brazil; shayra.kas@gmail.com (S.K.A.S.); brunnamartinsr@outlook.com (B.M.R.S.); 5 Rehabilitation Sciences, Graduate Program, Nove de Julho University (UNINOVE), São Paulo 01504-001, SP, Brazil; lucianamalosa@gmail.com; 6 Facultad de Ciencias de la Salud de Manresa, Universitat de Vic-Universitat Central de Catalunya (UVic-UCC), 08242 Manresa, Spain; 7 Health Sciences Graduate Program, Institute of Medical Assistance to State Public Servants (IAMSPE), Av. Ibirapuera, 981, São Paulo 04029-000, SP, Brazil; sergiorobertonacif@gmail.com; 8 Institute of Translational Pharmacology, National Research Council of Italy (CNR), 90146 Palermo, Italy; giuseppe.insalaco@ift.cnr.it

**Keywords:** COVID-19, functional status, health-related quality of life

## Abstract

Background: COVID-19 mainly affects the respiratory system, although its manifestations are multisystemic. We are increasingly recognizing complications that present after the acute phase, which are associated with impaired functional status and health-related quality of life (HRQoL). The objective was to assess the functional status and HRQoL of patients with post-COVID-19. Methods: This was a cross-sectional study involving individuals affected by COVID-19 who had persistent symptoms for one month after the acute phase of the disease. Functional status was measured with the six-minute walk test (6MWT), the Fatigue Severity Scale (FSS), the Medical Research Council (MRC) Dyspnea Scale, and the Post-COVID-19 Functional Status Scale (PCFS). HRQoL was confirmed with the Short-Form Health Survey 36 (SF-36). Results: We included 123 patients; 73 (59.35%) were male, with a mean age of 49.17 ± 13.48 years and a body mass index of 31.02 ± 6.56 stratified into three groups: the not-recovered group (NRG = 23), the ward-recovered group (WHG = 60), and the intensive-care-unit group (ICUG = 40). The main symptoms were muscle weakness (74.17%) and dyspnea (68.33%). The predicted distances for the 6MWT were missed by 12.83% by the GNR group, 20.21% by the GNR group, and 28.82% by the UGCI group. The MRC dyspnea scale had a mean value of less than 3, and the FSS scale had a mean value of over 4, indicating considerable fatigue. In the PCFS scale, a significant difference was observed (*p* < 0.0005), while in the SF-36, all HRQoL domains were compromised. Conclusion: Post-COVID-19 patients involved in this study showed a significant decline in functional status and an impairment of HRQoL.

## 1. Introduction

The coronavirus disease 2019 (COVID-19) infection, caused by severe acute respiratory syndrome coronavirus 2 (SARS-CoV-2), is the biggest health emergency around the globe in recent memory. Its manifestations are multisystemic, although the respiratory system is the main target of the disease [1]. At least 1 in 10 survivors of COVID-19 who had symptomatic infection before vaccination developed complications due to virus-specific physiological changes, compromised immune response, and inflammatory damage in response to acute infection [2,3].

The epidemiological data of COVID-19 vary by location and period, but due to the high index of asymptomatic infections, symptomatic cases do not reflect the total number of infected patients [4]. In mild COVID-19 cases, the symptoms are usually anosmia, ageusia, cough, fever, and muscle fatigue [5]. In severe cases, dyspnea and/or hypoxemia can rapidly progress to acute respiratory distress syndrome, difficult-to-correct metabolic acidosis, and coagulation dysfunction, along with changes in renal function, which have the potential to cause serious sequelae, especially in the lungs, or progress toward multiple organ failure and death [6,7].

In cases of patients with COVID-19 hospitalized in wards and intensive care units, the effects of long-term treatment or hospitalization resemble those of other severe infections, including the presence of post-intensive care syndrome (PICS), resulting in severe muscle weakness and the presence of post-traumatic stress disorder [7]. A recent systematic review with meta-analysis showed that patients hospitalized for COVID-19 exhibited post-acute COVID-19 symptoms, such as dyspnea, anxiety, and generalized myalgia when compared to those not hospitalized [8].

With increasing scientific evidence, a higher number of complications after the acute phase are being recognized and are being associated with increased morbidity, especially in patients who progressed to severe disease with thromboembolic complications associated with fatigue and dyspnea [9,10,11]. As per some reports, post-COVID-19 patients show a considerable presence of myalgia and arthralgia, impairment of lung function, physical performance, and acquired muscle weakness, which is associated with reduced functional status and compromised health-related quality of life (HRQoL) [12,13].

Identifying possible risk factors, defining diagnostic criteria, and understanding the severity and frequency of clinical and functional manifestations have become essential to ensure that preventive and therapeutic measures can be implemented in post-COVID-19 patients at the individual level based on this knowledge [14,15]. The aim of this study was to assess the functional status and HRQoL in patients with post-COVID-19 sequelae.

## 2. Materials and Methods

### 2.1. Study Design

A cross-sectional, observational study was performed at a single clinical and rehabilitation center involving individuals with persistent symptoms approximately a month after COVID-19 infection or hospital discharge, in which all patients underwent a clinical assessment, physical assessment, and the application of specific instruments to assess functional status and HRQoL. This study followed the guidelines of the Strengthening the Reporting of Observational Studies in Epidemiology (STROBE) [16].

**Figure 1:**
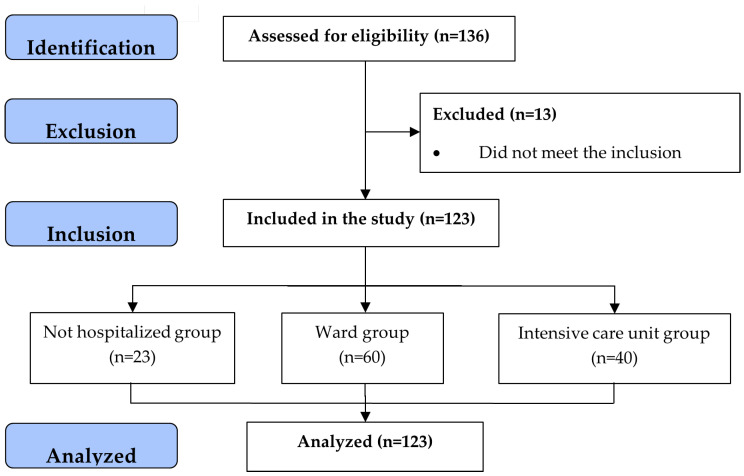
Flowchart of the study according to STROBE.

A total of 136 post-COVID-19 symptomatic patients were evaluated at admission to an outpatient Pulmonary Rehabilitation Program (PRP). Of these, three patients had neuromuscular conditions, nine patients were clinically unstable, and one patient had a recent unrecovered musculoskeletal injury that made it impossible for them to perform activities, being excluded from the sample, remaining 123 patients. In the sequence, for the analysis and comparison of the results, the population involved in this study was grouped into three groups. As shown in Figure 1, 23 patients as those who were not hospitalized but developed mild symptoms in the acute period of COVID-19 infection and had persistent symptoms and/or new complaints (not hospitalized [NHG group]). Another group consisted of 60 patients who presented with moderate symptoms and were admitted to a conventional hospital ward (hospitalized in the ward [WHG]), and the third group was 40 patients who developed severe symptoms and needed to be admitted to the ICU due to serious clinical complications (hospitalized in the ICU [ICUG]).

The team of researchers comprised pulmonologists and physiotherapists duly trained to carry out physical and functional assessments by applying specific, appropriate instruments. All assessments were performed by the same healthcare professionals involved in the study, with monitoring of vital signs during the tests. All assessment data were collected by the researchers and were recorded on standardized forms for each outcome; they were then entered into a Microsoft Excel database for further verification and analysis. The study was carried out at the Pulmonary Rehabilitation Laboratory of the Evangelical University of Goiás—UniEVANGÉLICA.

### 2.2. Ethical Aspects

This study was approved by the Research Ethics Committee (CEP) of Universidade Evangélica de Goiás—UniEVANGÉLICA (n° 4,296,707) and was also registered on ClinicalTrials.org (ID: COVID-19 PULMONARY REHAB NCT04982042). All patients involved in the study signed the Free and Informed Consent Form. Due to the fact that we are still in the acute phase of contagion, during all research activities, international biosecurity recommendations were followed to protect against infection caused by COVID-19.

### 2.3. Selection of Participants

Patient recruitment began in May 2021, with a continuous flow through social media and banners posted in referral hospitals for the treatment of patients with COVID-19 in the municipal, state, and private health network in the city of Anápolis (GO), Brazil.

### 2.4. Inclusion Criteria

In this study, adult patients, regardless of gender, aged between 18 and 75 years, with persistent symptoms or sequelae of COVID-19 infection confirmed by polymerase chain reaction or serology, who were seeking a rehabilitation program were included. We included patients who were clinically stable and agreed to participate in the study by signing the informed consent form. The clinical stability criterion was defined as no acute symptoms, no therapeutic variation, and no use of antibiotics and/or corticosteroids unless they were chronically used.

### 2.5. Exclusion Criteria

Patients with clinical instability (decompensated congestive heart failure, fever, systemic arterial hypertension, or acute rheumatoid disease), psychiatric and/or psychological disorders that compromised understanding, musculoskeletal disorders that prevented physical activities, and patients with neoplastic disease were excluded from the study. Patients who were unable to perform the proposed functional tests were also excluded.

### 2.6. Outcomes and Measurement Instruments

#### 2.6.1. Exercise Capacity: Six-Minute Test

The six-minute walk test (6MWT) is a safe, simple, reliable, and low-cost tool to assess exercise tolerance and functional capacity in patients with cardiorespiratory impairment, correlating excellently with morbidity and mortality [17]. In this study, the test was performed in a flat and rigid space of 30 m in length, while vital signs were monitored, including the scale of perceived exertion (lower-limb fatigue and dyspnea), according to guidelines published by the American Thoracic Society (ATS) [18]. To calculate the predicted values, the reference values for the healthy Brazilian population were used [19].

#### 2.6.2. Muscle Fatigue: Fatigue Severity Scale (FSS)

The FSS is a scale that is widely used to classify the severity of a patient’s symptoms of muscle fatigue through symptoms that interfere with motivation, performance, physical function, and activities of daily living (ADLs). It consists of a self-assessment instrument, validated in several languages, including Brazilian Portuguese, consisting of nine items ranging from one to seven in degrees of agreement [20,21]. Patients were instructed to respond to the scale based on the last seven days. The final score was obtained by dividing the average score by nine items, with higher scores indicating more pronounced fatigue and a score ≥ 4 already indicating the presence of fatigue [21].

#### 2.6.3. Severity of Dyspnea: Medical Research Council (MRC) Dyspnea Scale

The MRC is an instrument that assesses the sensation of dyspnea during ADLs, traditionally used in the international literature mainly because it is easy to apply and understand. The MRC scale is composed of five items, and the patient chooses the item that corresponds to how much the sensation of dyspnea limits their ADL [22]. In this study, the validated version in Brazilian Portuguese was used [23].

#### 2.6.4. Functional Status: Post-COVID-19 Functional Status Scale (PCFS)

PCFS is a simple tool proposed from the post-venous thromboembolism (VTE) Functional Status Scale, considering that post-COVID-19 patients have had recurrent complications mainly caused by venous thromboembolism; therefore, the assessment of functional limitations is considered relevant and useful [24].

Therefore, as it covers limitations in ADLs, the PCFS was adapted and validated to assess the impact of COVID-19 on functional status, and should be a supporting assessment tool to the others instead of a substitute. The scale presents a stratification of functional limitations ranging from Grade 1 to 4 and even Grade 5, which was left out in this study because it refers to “death”. In this study, patients were asked about their health status according to the last seven days [25].

#### 2.6.5. HRQoL: Short-Form Health Survey 36 (SF-36)

The SF-36 is a generic, multidimensional instrument that has been used to assess HRQoL in post-COVID-19 patients [26]. The questionnaire consists of 36 items that analyze eight domains related to the patient’s health: functional capacity, limitations due to physical aspects, pain, general health status, vitality, social aspects, limitations due to emotional aspects, and mental health, which are characterized under the physical and mental components. In order to calculate the scores, the SF-36 recommendations were followed so that for each domain, a final score from 0 to 100 was generated, and the higher the score, the better the HRQoL [27,28].

### 2.7. Statistical Analysis

The Kolmogorov–Smirnov test was used to test the normality of the distribution of the variables studied. For intragroup comparisons, the Student’s *t* test was used for paired samples that presented a parametric distribution or the Mann–Whitney U test for variables whose distribution was non-parametric. Intergroup comparisons were performed by one-way analysis of variance (ANOVA), and Tukey’s post-test was used for paired comparisons whenever the null hypothesis was rejected by ANOVA. Kruskal–Wallis test was used for intergroup comparisons of variables that presented with non-parametric distribution, and paired comparison (when the null hypothesis was rejected) was performed using the Mann–Whitney U test.

To evaluate the intragroup correlations, the Pearson correlation coefficient was used for samples that exhibited parametric distribution, and the Spearman correlation coefficient was used for samples that exhibited non-parametric distribution. All analyses were performed using SPSS version 21.0 for Windows (Chicago, IL, USA), and the significance level established for all analyses was 5%.

## 3. Results

In the three groups, 73 (59.35%) patients were male, 62 (50.41%) were of a mixed race, with a mean age of 49.17 ± 13.48 and a body mass index (BMI) of 31.02 ± 6.56. The other sociodemographic and clinical characteristics of the participants are shown in Table 1. The major self-reported symptoms presented by post-COVID-19 patients are described in Table 2. Considering the total number of patients involved in the study, the self-reported symptoms of muscle weakness (74.17%) and dyspnea (68.33%) stood out from the others; however, only muscle weakness was statistically significant between the groups (*p* < 0.05).

The main comorbidities prior to COVID-19 infections are shown in Table 3. A considerable percentage of patients had systemic arterial hypertension (SAH) and anxiety; its prevalence was based on the severity of the patients.

After the diagnosis of COVID-19, some patients developed clinical complications. Among them, one patient (4.35%) from the NHG group suffered an acute myocardial infarction (AMI), and another patient (4.35%) had a transient ischemic attack (TIA). In the WHG groups, two patients (3.33%) had an AMI, two (3.33%) had deep vein thrombosis (DVT), one patient (1.67%) had TIA, and two patients (3.33%) had a pulmonary embolism. In the ICUG group, two patients (5%) had AMI, one (2.5%) had DVT, another (1.67%) had a cerebrovascular accident, and two (5%) patients had pulmonary embolism.

Data regarding exercise capacity, dyspnea sensation, functional status, and muscle fatigue are described in Table 4. Patients who did not complete the 6MWT due to dyspnea and lower-limb muscle fatigue were excluded from the sample (four outpatients, eight hospitalized in the ward, and seven in the ICU). The distance covered in the 6MWT and its predicted values showed that outpatients walked 12.83% less than expected, those hospitalized in the ward walked 20.21%, and those admitted to the ICU walked 28.82% of the predicted distance for healthy subjects.

The assessment of the HRQoL, demonstrated in Table 5, showed that the patients included in the study had a worse performance in the ‘limitation by physical aspects’ domain (*p* < 0.01) in all three groups. The domains ‘general health status’ (*p* < 0.05) and ‘mental health’ (*p* < 0.05) were also significant, demonstrating that hospitalized patients had better rates than non-hospitalized patients.

**Figure 2:**
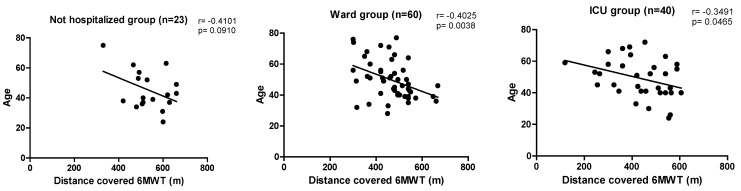

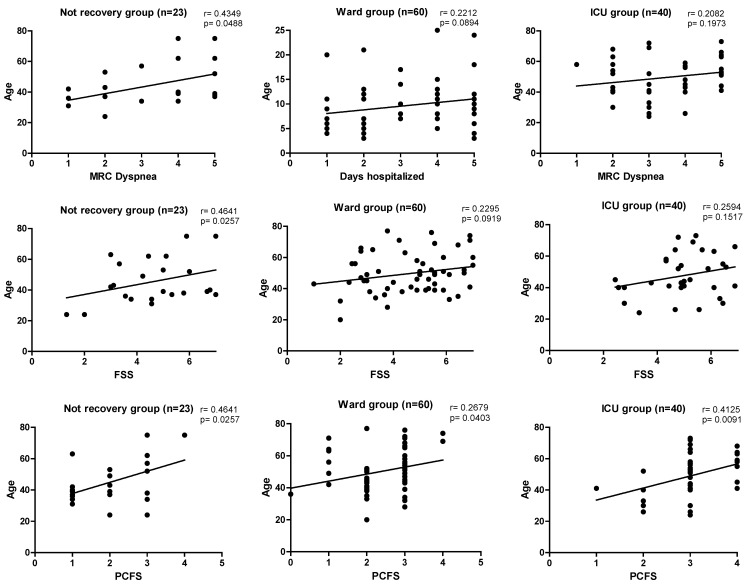
Main correlations observed between sociodemographic data and functional status. Note: 6MWT—six-minute walk test, m—meters, ICU—intensive care unit, FSS—Fatigue Severity Scale, MRC—Medical Research Council, PCFS—Post-COVID-19 Functional Status.

**Figure 3:**
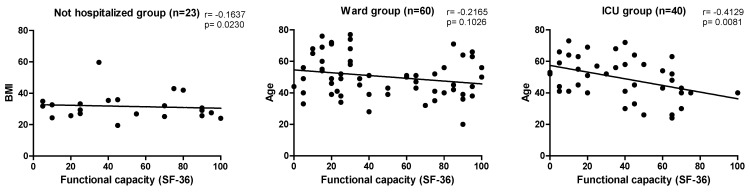

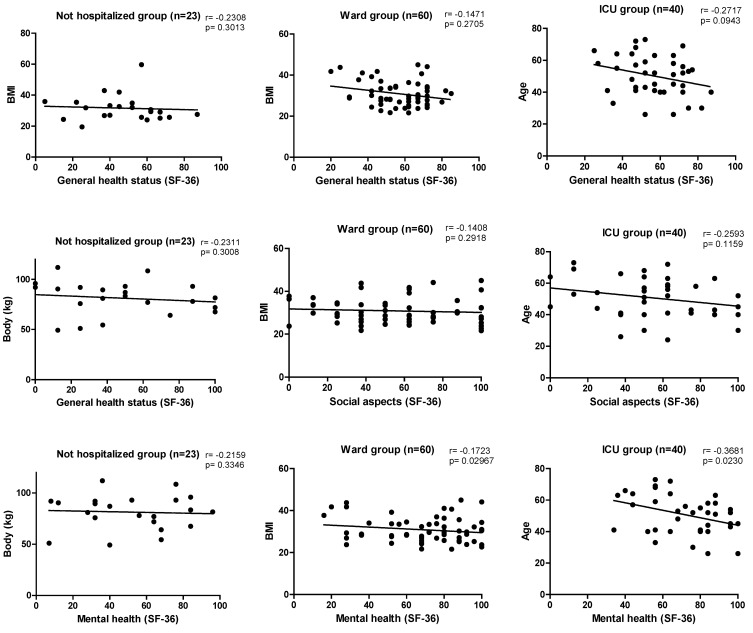
Correlations observed between sociodemographic data and health-related quality of life. Note: ICU—intensive care unit, BMI—body mass index, SF-36—Short Form Health Survey 36, kg—kilogram.

The main correlations observed regarding the outcomes that assessed functional status are shown in Figure 2, and those that measured HRQoL are found in Figure 3.

## 4. Discussion

The results of this study showed that in certain cases, the symptoms and alterations present were more pronounced in patients admitted to the ICU, followed by those in the ward, and in those not hospitalized, although some variables did not support this. The mean age of the patients involved in the study was 49.17 years, and the mean BMI value was 31. Most of them were male and of mixed ethnicity. The main symptoms observed were muscle weakness and dyspnea, which corroborates with the findings of a systematic review with a meta-analysis conducted in 2024 by Rochmawati, Iskandar, and Kamilah [11]. Li et al. also highlighted the presence of cough, dyspnea, myalgias, arthralgias, muscle fatigue, and generalized weakness as the main symptoms reported in the post-acute phase in mild-to-critical cases of COVID-19 [29].

In this study, the main comorbidities prior to COVID-19 infection were SAH, anxiety, and obesity, and these can become risk factors when they present multiple times in the same individual [30]. Several studies have shown that the main risk predictors associated with the severity of complications in infected patients, as well as in those with reduced functional status and HRQoL, were age, male gender, smoking, obesity, need and length of hospitalization, and use and length of mechanical ventilation during ICU stay [30,31,32]. In the present study, negative correlations were also observed between the patients’ ages and the scales and tests applied to assess functional status, such as the 6MWT, the FSS, the MRC dyspnea scale, and the PCFS scale in all groups, except for the WHG MRC dyspnea scale, which showed a positive correlation with the number of days hospitalized. Functional status is defined as a person’s level of autonomy and independence when performing ADLs [33]. The importance of assessing functional capacity in post-COVID-19 patients is highlighted, which can be perfectly carried out through the evaluation of exercise capacity using the 6MWT, considered the gold standard for various populations [34]. In this study, it was possible to observe that in the 6MWT, patients showed a significant difference in the distance covered in meters, 520.42 ± 93.89 (NHG), 450.03 ± 96.43 (WHG), and 420.73 ± 122.78 (ICUG), according to the distribution of the groups (*p* < 0.001) and in relation to the predicted values for the Brazilian population 87.17 ± 16.00 (NHG), 79.79 ± 13.92 (WHG), and 71.18 ± 18.74 (ICUG).

This demonstrated that the group hospitalized in the ICU had a worse physical performance as compared to the group hospitalized in the ward and sequentially to the non-hospitalized group (*p* < 0.001). The results shown in this present study corroborate the findings of the study by Lombardi et al. (2021), who stratified three groups of patients according to the worst PaO2/FiO2 (p/F) values obtained by arterial blood gas analysis during hospitalization, approximately one month after hospital discharge. The groups were divided into mild (p/F ≥ 300), moderate (≤200 p/F < 300) and severe (p/F < 200). The authors demonstrated that the greater the degree of compromised respiratory functions, the shorter the distance covered in the 6MWT in relation to patients with higher p/F, and were able to observe that patients with mild hypoxemia have lower exercise tolerance when compared to patients with severe hypoxemia (+80.0 m on 6MWT; *p* = 0.004) [35].

The study by Cortés-Telles et al. (2021) evaluated 186 patients who exhibited clinical and sociodemographic characteristics similar to those in this study. The patients were evaluated approximately one month after the onset of acute COVID-19 symptoms and divided into three groups. (51 leves; 26 moderados; 109 graves). While performing the 6MWT, patients with mild impairment walked a distance in meters greater than (493 ± 74) as compared to the other groups (428 ± 97/436 ± 111); however, when compared to the predicted percentage values, it was similar between the groups (83 ± 13; 82 ± 19; 83 ± 21, respectively), which differs from the findings of this present study in which the predicted values were proportional to severity [36]. Other cohort studies followed post-COVID-19 patients for a longer period of time after the infectious period and hospital discharge. These studies also demonstrated that over time, physical performance increases progressively [32,37,38,39]. However, even so, the distance traveled may remain below the predicted values, as verified in the study by Núñez-Cortés et al. (2022), where a positive correlation was found between the presence of multiple comorbidities and poorer performance in the 6MWT during the one-year follow-up assessments [40]. Thus, the 6MWT has proven to be an excellent tool for screening patients who exhibit impaired functional status and exercise capacity, especially those who have been hospitalized [34]. In this study, it was observed that the sensation of dyspnea evaluated by the dyspnea scale of the MRC presented a varied distribution between the degrees (1–5) but with similar means between the NHG and the patients of the WHG and ICUG (3.38 ± 1.56; 3.02 ± 1.46; and 3.44 ± 1.23, respectively), demonstrating that personal factors and the immune response to the virus can directly influence infected individuals. A study by Johnsen et al. (2021) evaluated 34 inpatients and 23 outpatients after three months of COVID-19 infection. The authors observed that 67% of the patients were symptomatic, presenting an MRC ≥ 2, with no difference between hospitalized and non-hospitalized patients, which is similar to the findings of our present study [41].

The functional status verified in this study through the PCFS presented an increasing average according to the severity in the outpatients to the hospitalized patients. Values of 2.17 ± 1.03 (NHG), 2.42 ± 0.79 (WHG), and 2.98 ± 0.77 (ICUG) were observed, showing statistical significance (*p* < 0.0005); 60 patients had moderate functional limitations (grade 3) representing 48.78% of the sample, and 33 patients (26.83%) had mild functional limitations.

A cohort of 43 patients with COVID-19 was followed for one year and was evaluated at hospital discharge, 3 months, and 12 months after admission. Of these patients, 10 (23%) had mild pneumonia, 17 (40%) moderate, 10 (23%) severe, and 6 (14%) critical. It was observed in the study that 8 out of 34 patients had reduced physical performance assessed by the 6MWT, 9 of 34 were classified as ≥2 on the mMRC dyspnea scale, 14 of 32 had functional limitations on the PCFS scale, and 9 of 42 patients had reduced HRQoL [38].

In a study involving 444 patients evaluated between four and eight weeks after hospital discharge due to complications from COVID-19, it was observed that 80% of the sample presented different degrees of functional alterations, ranging from very mild (63.1%), mild (14.4%), moderate (2%), and severe (0.5%), assessed by PCFS [42]. In the study by Giurgi-Oncu et al. (2021), 82 patients (57.34%) were evaluated by PCFS during the first 6 weeks. Of these 48 inpatients and 34 outpatients, 15 (18.29%) were classified as Grade 1, presenting very mild limitations, 35 (42.68%) as Grade 2 that was equivalent to mild limitations, and 32 (39.02%) as Grade 3 with moderate limitations [43].

Muscle fatigue verified by the FSS scale in this present study was not significant between hospitalized and non-hospitalized patients, although both groups had an index of <4, which is already characterized as the presence of fatigue. Few studies using the FSS were found in the literature, and one of these studies had a sample composed of 206 adult patients who were hospitalized from COVID-19 infection and were evaluated between four to six weeks after discharge. Of the 206 patients, 126 (61.2%) had at least one symptom of fatigue according to the FSS, with a mean FSS score of 32.1 ± 15.28, and the mean level of overall fatigue was 5.93 ± 2.90 [44].

The study by Eleftheriou et al. (2021) evaluated 20 patients approximately five months after infection with COVID-19. Of these, 12 had been hospitalized. Among patients who completed the FSS, 13 (85%) had a significant level of fatigue, and the raw score was 48.9 ± 16.8 [45]. A cohort study conducted by AlRasheed et al. in 2023 followed post-COVID-19 patients for a period of 6 to 24 months, assessing the severity of fatigue compared to a control group. The authors demonstrated that the FSS scores showed significantly higher results 3 (1.8–4.3) in post-COVID-19 patients compared to the control group 2.6 (1.4–4) (*p* < 0.001). It was also observed that these scores correlated negatively with the physical and mental domains of the SF-36 [46]. HRQoL refers to the level of well-being perceived by the individual in the various domains of their life, considering their impact on general health. The measurement of quality of life is somewhat subjective due to the patient’s difficulty in correlating changes with the multiple areas of their life [47]. However, in the last few studies, specific instruments have been used to assess the HRQoL, thus reducing the individual subjectivity of each patient. The evaluation of HRQoL covering physical, psychological, and social factors through specific instruments is of great importance to determine the prognosis of patients who have been infected by COVID-19 [48].

In the present study, the assessment of HRQoL was significant between the groups in the domain ‘limited by physical aspects’, ‘general health status’, and ‘mental health’ (*p* < 0.05), but it is worth noting that the last two domains mentioned were inversely proportional, demonstrating that hospitalized patients and, consequently, those with a more severe clinical condition, had a better perception of general and mental health, which may be associated with satisfaction of having received proper attention and care necessary for the treatment of COVID-19. In this study, negative correlations were also found between age, weight, and BMI among the three groups in the domains of “functional capacity”, “general health status”, “social aspects”, and “mental health”. A study by Elber et al. (2021) involved 18 patients with COVID-19 who required intensive care, evaluated after hospital discharge at an average of 36, 75.5, 122, and 222 days. The HRQoL measured by the SF-36 showed that the physical component had a greater reduction, improving over time, but when compared to the reference groups, they remained impaired. The most accentuated alterations were in the first moment of evaluation in the domain ‘limitation by physical aspects’ (16.1 ± 31.9) and ‘functional capacity’ (33.3 ± 31.7), which agrees with the findings of this study. When comparing the expected values in healthy people, the domain ‘social aspects’ (60.7 ± 27.2), ‘limitations by emotional aspects’ (58.3 ± 47.4), and ‘general health’ (51.8 ± 13.5) were more compromised [26].

In this way, it can be said that in post-COVID-19 patients who were followed up between 3 to 12 months, a reduction in exercise capacity was observed, as well as limitations in HRQoL, with a greater impact on physical components, results that demonstrate an interconnection with the severity of fatigue [49,50]. There is a need for post-COVID-19 patients, especially those hospitalized, to be referred to a clinical for functional and psychosocial evaluation in order to identify the changes arising in the post-infection period [37]. In this sense, with a focus on helping to manage follow-up strategies, an interesting systematic review mapped scales and tests that assess physical performance in this population [51]. With this review, the authors observed that a wide variety of functional status tests have been performed, making comparisons between studies difficult; however, all studies involved in the systematic review showed impairment in physical performance in post-COVID-19 patients. However, the quality of most studies was judged to be low or fair.

The findings of this study underscore the critical importance of monitoring patients after COVID-19 to assess the extent of functional impairment and HRQoL, particularly in those with severe cases who require hospitalization in a ward or ICU to manage complications. Despite these insights, the present study has certain limitations that should be noted, including its cross-sectional design and single-center scope, which may restrict the generalizability of the findings to other populations and settings. Moreover, the lack of longitudinal analysis hinders a deeper understanding of the progression of functional conditions and HRQoL over time. Future studies with longitudinal designs and more diverse samples are necessary to validate and extend the conclusions presented here.

## 5. Conclusions

Post-COVID-19 patients enrolled in this study showed a significant decline in functional status and an impairment in HRQoL. The impacts were more evident in hospitalized patients, especially those who required care in the intensive care unit (ICU). In this study, a significant limitation in functional capacity assessed by the 6MWT was observed, associated with the severity of the clinical condition during the infection phase. The PCFS scale revealed a higher frequency of moderate functional limitations in hospitalized patients, while the perception of fatigue assessed by the FSS was elevated in all groups, indicating significant muscle fatigue. Dyspnea, measured by the MRC scale, presented varying degrees of severity, suggesting that both individual factors and the immune response influence respiratory impairment. HRQoL was more impaired in the physical and general health domains, especially in patients with a history of ICU admission and higher BMI. Paradoxically, the hospitalized group showed a more positive perception regarding mental health, possibly associated with satisfaction with the care received during hospitalization. Such findings highlight the need for early and continuous rehabilitation interventions to minimize functional sequelae and improve quality of life, with individualized strategies based on the severity and specific limitations of each patient.

## Figures and Tables

**Table 1:** Sociodemographic and clinical characteristics presented by post-COVID-19 patients.

Variables	Not Hospitalized (*n* = 23)	Hospitalized		*p*
		Ward (*n* = 60)	ICU (*n* = 40)	
Sex				ns
Male	12 (52.17%)	34 (56.67%)	27 (67.5%)	
Feminine	11 (47.83%)	26 (43.33%)	13 (32.6%)	
Ethnicity				ns
White	8 (34.78%)	23 (38.33%)	13 (32.5%)	
Brown	11 (47.83%)	28 (46.67%)	23 (57.5%)	
Black	4 (17.39%)	9 (15.00%)	4 (10%)	
Age (years)	45.78 ± 15.25	50.32 ± 12.97	49.48 ± 13.18	ns
Weight (kg)	85.61± 24.02	85.49 ± 16.7	87.1 ± 20.59	ns
BMI	31.42 ± 8.34	30.84 ± 5.9	31.06 ± 6.52	ns
SBP (mmHg)	125.45 ± 16.54	121.87 ± 12.31	120.5 ± 13.39	ns
DBP (mmHg)	81.36 ± 15.21	81.00 ± 10.69	80.25 ± 12.09	ns
Hospitalization time (days)	N/A	9.48 ± 4.94	18.26 ± 9.42	***
ICU stay (days)	N/A	N/A	11.97 ± 9.36	
Oxygen therapy	2 (8.70%)	60 (100%)	40 (100%)	ns
NIV	N/A	30 (50%)	40 (100%)	***
IMV	N/A	N/A	12 (20%)	

Note: ICU: intensive care unit; BMI: body mass index; kg: kilo; mmHg: millimeter of mercury; SBP: systolic blood pressure; DBP: diastolic blood pressure; N/A: not applicable; NIV: non-invasive ventilation; IMV: invasive mechanical ventilation; ns: not significant; ***: *p* < 0.0005.

**Table 2:** Main symptoms presented by post-COVID-19 patients.

Variables	Not Hospitalized (*n* = 23)	Hospitalized		*p*
		Ward (*n* = 60)	ICU (*n* = 40)	
Ageusia	4 (17.39%)	13 (21.67%)	9 (22.5%)	ns
Anosmia	5 (21.74%)	12 (20%)	8 (20%)	ns
Changes in sleep	15 (65.22%)	30 (50%)	19 (47.5%)	ns
Visual changes	4 (17.39%)	10 (16.67%)	6 (15%)	ns
Arthralgia	5 (21.74%)	15 (25%)	12 (30%)	ns
Headache	14 (60.87%)	26 (43.33%)	19 (47.5%)	ns
Concentration deficit	4 (17.39%)	9 (15%)	4 (10%)	ns
Memory deficit	13 (65.52%)	25 (41.67%)	10 (25%)	ns
Balance deficit	9 (39.13%)	23 (38.33%)	19 (47.5%)	ns
Dyspnea	17 (73.91%)	39 (65%)	29 (72.5%)	ns
Muscle weakness	14 (60.87%)	41 (68.33%)	36 (90%) ^a,b^	*
Myalgia	12 (52.17%)	25 (41.67%)	19 (47.5%)	ns
Paresthesia	10 (43.48%)	16 (26.67%)	13 (32.5%)	ns
Tachycardia	12 (52.17%)	29 (48.33%)	18 (45%)	ns
Tremors	9 (39.13%)	23 (38.33%)	14 (35%)	ns
Dizziness	13 (65.52%)	22 (36.67%)	13 (32.5%)	ns
Cough	6 (26.09%)	30 (50%)	23 (57.5%)	ns

Note: ICU: intensive care unit; ns: not significant; *: *p* < 0.05. ^a^: statistically significant difference between the ‘hospitalized in the ward’ group and the ‘hospitalized in the ICU’ group; ^b^: statistically significant difference between the ‘non-hospitalized’ group and the ‘hospitalized in the ICU’ group.

**Table 3:** Main comorbidities presented by patients prior to COVID-19 infection.

Variables	Not Hospitalized (*n* = 23)	Hospitalized		*p*
		Ward (*n* = 60)	ICU (*n* = 40)	
Anxiety	7 (30.43%)	13 (21.67%)	14 (35%)	ns
Asthma	1 (4.35%)	3 (5%)	0 (0%)	ns
Depression	2 (8.7%)	6 (10%)	2 (5%)	ns
Dyslipidemia	4 (17.39%)	6 (10%)	4 (10%)	ns
Diabetes Mellitus	1 (4.35%)	5 (8.33%)	9 (22.5%) ^b^	**
COPD	1 (4.35%)	2 (3.33%)	0 (0%)	ns
Hypothyroidism	2 (8.0%)	6 (10%)	1 (2.5%)	***
Hepatic steatosis	2 (8.7%)	2 (3.33%)	3 (7.5%)	ns
SAH	5 (21.74%)	17 (28.33%) ^a^	18 (45%) ^b^	ns
Obesity	2 (8.7%)	12 (20) ^a^	10 (25%) ^b^	**

Note: ICU: intensive care unit; SAH: systolic arterial hypertension; COPD: chronic obstructive pulmonary disease; ns: not significant; **: *p* < 0.005; ***: *p* < 0.0005. ^a^: statistically significant difference between the ‘non-hospitalized’ group and the ‘hospitalized in the ward’ group; ^b^: statistically significant difference between the ‘non-hospitalized’ group and the ‘hospitalized in the ICU’ group.

**Table 4:** Exercise capacity, sensation of dyspnea, functional status, and muscle fatigue in post-COVID-19 patients.

Variables	Not Hospitalized (*n* = 23)	Hospitalized		*p*
		Ward (*n* = 60)	ICU (*n* = 40)	
6MWD’ (m)	520.42 ± 93.89	450.03 ± 96.43 ^a^	420.73 ± 122.78 ^b^	**
6MWD’ pred (%)	87.17 ± 16.00	79.79 ± 13.92 ^a^	71.18 ± 18.74 ^b^	**
MRC dyspnea	3.38 ± 1.56	3.02 ± 1.46	3.44 ± 1.23	ns
Grade 1	6 (26.09%)	11 (18.33%)	1 (2.5%)	
Grade 2	3 (13.04%)	15 (25%)	10 (25%)	
Grade 3	2 (8.70%)	9 (15%)	10 (25%)	
Grade 4	5 (21.74%)	12 (20%)	8 (20%)	
Grade 5	7 (30.43%)	13 (21.67%)	11 (27.5%)	
PCFS	2.17 ± 1.03	2.42 ± 0.7 ^a^	2.98 ± 0.7 ^b^	***
Grade 1	8 (34.78%)	6 (10%)	2 (5%)	
Grade 2	5 (21.74%)	22 (36.67%)	6 (15%)	
Grade 3	8 (34.78%)	29 (48.33%)	23 (57%)	
Grade 4	2 (8.70%)	2 (3.33%)	9 (22.5%)	
FSS	4.77 ± 1.55	4.24 ± 1.78	4.87 ± 1.36	ns

Note: ICU: intensive care unit; 6MWD’: distance covered in the six-minute walk test; m: meters; pred: predicted; MRC: Medical Research Council; PCFS: Post-COVID-19 Functional Status; FSS: Fatigue Severity Scale; ns: not significant; **: *p* < 0.005 ***: *p* < 0.0005. ^a^: statistically significant difference between the ‘non-hospitalized’ group and the ‘hospitalized in the ward’ group. ^b^: statistically significant difference between the ‘non-hospitalized’ group and the ‘hospitalized in the ICU’ group.

**Table 5:** Health-related quality of life in post-COVID-19 patients.

Variables	Not Hospitalized (*n* = 23)	Hospitalized		*p*
		Ward (*n* = 60)	ICU (*n* = 40)	
SF-36 (0–100)				
Functional capacity	50.23 ± 32.42	47.29 ± 31.93	37.63 ± 25.7	ns
Limitation by physical aspects	19.32 ± 35.3	27.59 ± 38.81	9.38 ± 26.97 ^b,c^	*
Pain	53.23 ± 27.21	60.98 ± 30.74	54.50 ± 29.11	ns
General health status	46.95 ± 20.12	57.53 ± 14.49 ^a^	56.53 ± 15.35 ^c^	*
Vitality	45.23 ± 24.08	56.29 ± 26.08	55.75 ± 20.77	ns
Social aspects	47.73 ± 32.88	55.17 ± 30.17	54.44 ± 28.43	ns
Emotional aspects	43.94 ± 41.64	51.72 ± 43.79	44.16 ± 42.96	ns
Mental health	54.36 ± 27.09	69.47 ± 23.31 ^a^	70.95 ± 19.48 ^c^	*

Note: ICU: intensive care unit; SF-36: Short Form Health Survey 36; ns: not significant; *: *p* < 0.05. ^a^: statistically significant difference between the ‘not hospitalized’ group and the ‘hospitalized in the ward’ group. ^b^: statistically significant difference between the ‘hospitalized in the ward’ group and the ‘hospitalized in the ICU’ group; ^c^: statistically significant difference between the ‘non-hospitalized’ group and the ‘hospitalized in the ICU’ group.

## Data Availability

The raw data supporting the conclusions of this article will be made available by the authors upon request.
